# Tobacco and alcohol cessation or reduction interventions in people with oral dysplasia and head and neck cancer: systematic review protocol

**DOI:** 10.1186/s13643-017-0555-y

**Published:** 2017-08-10

**Authors:** Ellie Shingler, Luke A. Robles, Rachel Perry, Chris Penfold, Andy Ness, Steve Thomas, J. Athene Lane, Richard M. Martin

**Affiliations:** NIHR Bristol BRC Nutrition Theme, University Hospitals Bristol Research & Education Centre, Level 3, Upper Maudlin Street, Bristol, BS2 8AE UK

**Keywords:** Head and neck cancer, Oral dysplasia, Systematic review, Tobacco cessation, Alcohol

## Abstract

**Background:**

Head and neck cancers include malignancies of the mouth, larynx and oropharynx. Tobacco use and alcohol consumption are associated with increased risks of developing and dying from head and neck cancer. The aim of this review is to examine the effectiveness of smoking and alcohol cessation interventions on disease-related outcomes, quality of life and behavioural change in adults with head and neck cancer and oral dysplasia.

**Methods:**

The Cochrane library, CINAHL, Embase, MEDLINE, PsycINFO and Web of Science databases will be searched for randomised controlled trials investigating the effects of smoking or alcohol interventions on patients with either head and neck cancer or oral dysplasia. The primary outcomes are disease-free survival and, for participants with oral dysplasia, malignant transformation to cancer. Secondary outcomes are disease recurrence and progression, quality of life and behavioural change. The quality of included studies will be assessed using the ‘Cochrane Collaborations tool for assessing risk of bias’. A qualitative synthesis of the results will be reported, and a meta-analysis of the outcome data conducted, where appropriate.

**Discussion:**

This systematic review will identify the extent of the current research on smoking and alcohol cessation interventions in patients with head and neck cancer and oral epithelial dysplasia. The findings have the potential to inform which interventions have been successful and how future behavioural change interventions should be conducted within these populations.

**Systematic review registration:**

PROSPERO CRD42016038237

**Electronic supplementary material:**

The online version of this article (doi:10.1186/s13643-017-0555-y) contains supplementary material, which is available to authorized users.

## Background

Head and neck cancers are a heterogeneous group of malignancies which include cancers of the mouth, sinus, larynx, nasopharynx and oropharynx. The most common type of head and neck cancer is squamous cell carcinoma of the mucosal surfaces of the mouth, nose and throat [1] which accounts for 90% of cancers of the head and neck [2]. It is estimated that 11,152 new cases were diagnosed in the UK in 2012 [3], with more than 550,000 cases diagnosed annually worldwide [4]. Incidence rates vary based on cancer site and while rates of oral cavity and oropharyngeal cancers have risen over recent years, the incidence of laryngeal cancer rates has declined which may be related to the reduction in smoking rates [5]. European relative survival rates for head and neck cancer patients are estimated at 72% at 1 year and 42% at 5 years [6]. Overall 5-year survival for head and neck cancer in the UK has been estimated at 45%; however, rates also vary by cancer site [7]. In England, between 2009 and 2013, 5-year survival rates were 65.6% in oropharyngeal cancer, 56.1% in oral cavity cancer and 27.8% in hypopharyngeal cancer [8].

Oral epithelial dysplasia is a potentially pre-malignant condition that can progress to squamous cell carcinoma. Reported rates of mutation to oral cancer vary based on the degree of dysplasia (16% for sever dysplasia to <5% for mild dysplasia) [9]. Leukoplakia and erythroplakia are two of the most common clinical presentations in which dysplasia is found and rates of dysplasia vary between these lesions [10]. It is estimated that 5% of people with leukoplakia have dysplasia or cancerous cells while up to 50% of people with erythroplakia will develop cancer [11].

Tobacco use, which can include both smoking and chewing tobacco, and alcohol consumption are estimated to account for approximately 75% of cancers of the oral cavity, pharynx and larynx [12, 13]. In a pooled analysis [12] of 15 case control studies of cigarette smoking amongst study participants who never drank alcohol, smoking was associated with an increased risk of head and neck cancer (OR for ever versus never smoking = 2.13, 95% CI = 1.52 to 2.98) and a dose-response relationship was observed for the frequency and duration of cigarette smoking. The pooled analysis also reported that at least three alcoholic drinks per day amongst study participants who never smoked was associated with a 2.04 increased risk of head and neck cancer (95% CI = 1.29 to 3.21).

Conversely, the risk factors for oral dysplasia are not well understood; although, there is also evidence of links to tobacco use and alcohol consumption [14, 15].

As well as increasing risk of disease, there is also evidence to suggest that smoking and alcohol use can influence treatment outcomes [16, 17]. Smokers may be less likely to respond to treatment if they smoke, resulting in a lower rate of survival [18], and are at greater risk of experiencing treatment side effects [19], while patients who continue to consume alcohol seem to have a higher risk of recurrence and second primary tumours [20, 21].

No previous systematic reviews on smoking or alcohol interventions in patients being treated for head and neck cancer or oral dysplasia were identified through an initial scope of the literature, highlighting a need for the synthesis of intervention data in this area.

### Research objective

To examine the effectiveness of smoking and alcohol cessation interventions on disease-related outcomes, quality of life and behavioural change in adults with head and neck cancer and oral dysplasia.

## Methods

This protocol has been reported in accordance with the “Preferred Reporting Items for Systematic review and Meta-Analysis Protocols (PRISMA-P) 2015 checklist” [22]. A populated checklist for this review protocol has been provided in Additional file 1.

### Eligibility criteria

#### Study design

Randomised controlled trials will be included. Quasi-randomised trials, those where randomisation is done on the basis of a pseudo-randomised sequence [23], and observational studies will be excluded.

#### Participants

The participants include adult patients aged at least 18, diagnosed with either oral dysplasia or head and neck cancer. For the purpose of this review, head and neck cancers that will be included are cancers of the oral cavity, larynx, hypopharynx and oropharynx as these are associated with tobacco and alcohol use.

#### Intervention

Interventions that will be included in the review are as follows:

- Tobacco use reduction or cessation interventions—including both psychosocial and pharmacological interventions.
- Alcohol use reduction or cessation interventions—including both psychosocial and pharmacological interventions.

Trials that include a combination of these interventions will also be included.

#### Comparison

We will include placebo or standard care as the comparison group, depending on intervention type (pharmacological or behavioural). Studies that compare multiple active intervention arms but do not include a control arm will also be included.

#### Outcomes

Studies will be included in the data extraction if they report on one of the following primary or secondary outcomes.

Primary outcomes

1. *Disease*-*free survival*: defined as the length of time following treatment that the participant lived without recurrence or relapse of the disease.
2. *Malignant transformation* in participants with oral dysplasia: defined as the development of oral or laryngeal cancer.

Secondary outcomes

1. *Disease recurrence*: defined as the return of the cancer to the same primary site following treatment.
2. *Disease progression* in people with head and neck cancer: defined as the development or progression of lymph node metastasis, the development of distant metastasis or an increase in the size of the primary tumour.
3. *Quality of life* measured by any standardised scale: For example, including but not limited to the European Organisation for Research and Treatment of Cancer scales (including the head and neck specific scale “QLQ-H&N35”), Functional Assessment of Chronic Illness Therapy Measurement Systems, Short Form Health survey—SF36/SF12.
4. *Adverse events* related to the interventions. These could include but are not limited to nicotine/alcohol withdrawal related adverse events and adverse events related to nicotine replacement therapy.
5. *Behavioural change*: defined as the proportion of participants who successfully altered the behaviour of interest, measured through either self-report or biomarker methods.
6. *Second primary cancers* if they originated at least 3 cm away from the primary site and occurred at least 3 years after the last known recurrence
7. *Head and neck cancer specific mortality*
8. *All-cause mortality*

### Search strategy

#### Electronic searches

Literature searches will be performed in the following databases for relevant published articles:

1. Cochrane library (from inception to present)
2. PsycINFO (from 1806 to present)
3. CINAHL (from 1937 to present)
4. Embase (from 1974 to present)
5. MEDLINE (from 1946 to present)
6. Web of Science (from 1900 to present)

An example of the search strategy for use in MEDLINE is shown in Additional file 2. The same search terms will be used for each database with any changes made to the syntax as per individual database requirements. The searches will not be limited by date range or language of publication. Searches performed in Web of Science will enable conference abstracts to be yielded.

### Searching other resources

Any systematic reviews found will be used to identify further studies not found in the original search. The reference lists of all included articles will be hand searched for additional studies. The search will be complemented by contacting subject experts about any unpublished or published studies that were not yielded from the original search. The NIHR Clinical Trials gateway will also be searched to identify any trials currently taking place which have not yet reached publication stage [24] and the first 20 pages of google scholar will be hand searched for any additional articles.

### Data management

Endnote reference management software will be used to manage the search results. Database search results will be imported into an Endnote library and duplicates removed during the data screening process. All references to the same study will be extracted and referred to in the text of the main report.

### Selection of studies

Studies for inclusion will be screened independently by two reviewers (ES, LR). Initial screening will be performed by both reviewers and compared to ensure accuracy. Any discrepancies found will be discussed with a third reviewer (RP) for resolution. Abstracts meeting the inclusion criteria will be retrieved in full for data extraction.

### Data extraction

Data will be extracted by two reviewers independently, then compared for accuracy. Extraction will take place using a standardised template designed specifically for this review. This template will be piloted by both reviewers on the first five papers extracted, then discussed to ensure common use and to agree on any amendments required. Data will be extracted on the following:

- Publication information—paper title, author details, publication type, funding sources, year of study
- Sample characteristics—number of participants, demographics (age, sex, ethnicity), cancer site and staging, inclusion/exclusion criteria, sub groups included, withdrawals and exclusions
- Intervention type and design—study design, intervention description (including type, dose and duration), method of randomisation. Timing of the intervention (e.g. pre or post treatment) will also be extracted.
- Results—outcomes of interest reported (as defined in the methods section of this protocol), statistical methods used, summary statistics, means and standard deviations or medians and inter quartile ranges as appropriate, QOL scales used and resulting QOL scores, adverse events.
- Measures of methodological quality—detail provided below under assessment of risk of bias.

Any discrepancies found will be discussed with a third reviewer in order to finalise inclusion/exclusion.

### Assessment of risk of bias

Risk of bias and overall methodological quality will be assessed by two reviewers independently using a table based on the Cochrane Collaboration’s tool for assessing risk of bias, which has been updated to reflect the current study’s review parameters [25]. A copy of the table of assessment criteria used for this study can be seen in Table 1. Studies will be classified as having a low, high or unclear risk of bias based on six criteria:

- Sequence generation
- Allocation concealment
- Blinding of participants, personnel and outcome assessors (where practical and applicable)
- Incomplete outcome data
- Selective outcome reporting
- Other potential threats to validity, e.g. differences in baseline characteristics between intervention groups and conflict of interest of researchers.

**Table 1:** Risk of bias assessment table for the systematic review of smoking and alcohol interventions in head and neck cancer

Domain	Low risk	High risk
Sequence generation	Describes a random component, e.g. random number generator, coin toss and shuffling envelopes.	Includes a systematic, non-random component, e.g. hospital record number, DOB, participant preference and based on lab results.
Allocation concealment	Participants/investigators could not foresee allocation, e.g. central allocation system (web or phone based), sequentially numbered supplement/placebo containers and sequentially numbered sealed envelopes.	Participants/investigators could foresee allocation, e.g. an open random allocation schedule, DOB, case record numbers or assignment envelopes without appropriate safeguards.
Blinding of participants, personnel and outcome assessors	No blinding conducted but this is deemed not likely to outcome or outcome measurement. No blinding but this is deemed either not appropriate or possible, e.g. not blinding participants in PA or non-supplement-based dietary intervention. Blinding of participants and study personnel where appropriate	No/incomplete blinding where the outcome measurement is likely to have been affected. Blinding conducted but likely that the blinding could have been broken.
Incomplete outcome data	Reason for missing outcome data unlikely to be related to true outcome. If participants are reported and analysed in the groups they were allocated to irrespective of non-compliance/drop out. Up to 10% loss through drop out or loss-to follow-up.	Reason for missing outcome data likely to be related to true outcome. Participants are not analysed based on the group they were allocated.
Selective outcome reporting	If all pre-specified outcomes have been reported on.	Reporting on outcomes that were not pre-specified. Outcomes of interest are reported incompletely Fails to include results for an expected key outcome.
Other sources of bias	The study appears to be free of any other sources of bias.	Other possible cause of bias, e.g. extreme baseline imbalance, claim of fraudulence and conflict of interest for investigators not addressed.

### Dealing with missing data

Study authors will be approached for any missing or unreported data, as required. For the purposes of meta-analyses where missing data are still present, we will attempt to estimate these values from other results reported in the paper (e.g. if *t* values from *t* tests are reported but no standard deviations, we can calculate the standard deviations), or impute possible values using the largest values from those studies which did report the missing result (see “Sub-group and sensitivity analyses” for further details).

### Data analysis

We will undertake a qualitative synthesis of all studies. Due to the diverse nature of the interventions of interest, we anticipate heterogeneity between studies and will therefore use random effects models to quantitatively synthesise all data relating to each combination of intervention type and outcome measure. For binary outcome measures (disease-free survival, progression and recurrence, cancer specific and all-cause mortality, and second primary cancers), we will pool risk ratios and will calculate or estimate risk ratios where possible from studies which do not report them. Where hazard ratios are reported, we will pool these separately and we will estimate hazard ratios from studies which report suitable outcome measures (e.g. median survival time for intervention and control arms). For continuous outcomes (quality of life), we will pool means and associated standard deviations and we will estimate mean and standard deviation values where possible (e.g. median quality of life for intervention and control arms). We will assess statistical heterogeneity by visually inspecting forest plots and with the chi-square measurement, with a cutoff of *P* < 0.01 for the chi-square measurement to indicate heterogeneity since it is difficult to assess when sample sizes are small. We will use the *I* ^2^ statistic to measure variation in the effect size due to heterogeneity, with values greater than 50% indicative of significant heterogeneity [19]. If the studies are considered too heterogeneous with regards to the intervention design or outcome measure used, we will not pool their effect sizes. We will test the likelihood of publication bias through visual inspection of funnel plots and using Egger’s regression test.

If a meta-analysis is undertaken, the strength of the body of evidence will be assessed using the GRADE system [23].

Interventions which include a behavioural change technique will also be coded for qualitative analysis using the BCT Taxonomy (v1) to allow for comparison of techniques employed for the intervention [26].

### Sub-group and sensitivity analyses

Where possible, we will analyse intervention effects on primary and secondary outcomes within the following sub-groups: oral dysplasia compared with head and neck cancer and between tumour sites. To minimise the impact of studies at high risk of bias, we will exclude these and repeat our meta-analyses. Imputation of missing values using the largest reported value from other studies can potentially bias results towards a lack of effect [23], and therefore, as sensitivity analyses to explore the impact of this imputation method on our findings, we will repeat our meta-analyses with two modifications. Firstly, instead of replacing missing values with the largest reported values, we used the mean of the reported values. Secondly, studies which were missing data were excluded.

## Discussion

Due to evidence from observational studies that suggests that those who reduce tobacco and alcohol consumption have better outcomes, lifestyle interventions aimed at cessation are of current interest. However, to our knowledge, no systematic review has been published to date that identifies the interventions tested or how successful they have been at improving clinical outcomes and quality of life. We anticipate that this review will therefore inform the design and conduct of further behavioural change interventions in this population group.

This systematic review will identify the extent of the current research on smoking and alcohol cessation interventions in patients with head and neck cancer and oral dysplasia. Through review of the current evidence, the findings will inform further research into interventions aimed at improving outcomes and quality of life in this patient group.

## Additional files

Additional file 1: PRISMA-p checklist for the reporting of the protocol. (DOC 82 kb)

Additional file 2: An example of the search terms used in MEDLINE. (DOCX 17 kb)

## Abbreviations

BCT: Behavioural change technique
GRADE: Grading of Recommendations, Assessment, Development and Evaluation
OED: Oral epithelial dysplasia
PA: Physical activity
QOL: Quality of life
RCT: Randomised controlled trial
