# Supplementary material for: Tobacco and alcohol cessation or reduction interventions in people with oral dysplasia and head and neck cancer: systematic review protocol
Source: Syst Rev. 2017 Aug 10;6:161. doi: 10.1186/s13643-017-0555-y (PMC5551025; doi:10.1186/s13643-017-0555-y)
Supplement: Supplementary file 2 — An example of the search terms used in MEDLINE. (DOCX 17 kb) [file 13643_2017_555_MOESM2_ESM.docx]

**Additional File 2 - MEDLINE search via OVID, june 2016**

| 1. (head and neck neoplasms).mp. [mp=title, abstract, original title, name of substance word, subject heading word, keyword heading word, protocol supplementary concept word, rare disease supplementary concept word, unique identifier] |  |
| --- | --- |

| 2. ((oral or mouth or head or neck or oesophag* or esophag* or pharynx or pharyngeal or aerodigestive) adj5 (neoplas* or cancer* or carcinoma* or tumo?r*)).tw. |  |
| --- | --- |

| 3. ((larynx or laryngeal* or face or facial or tongue* or lip* or hypopharyn* or buccal or tonsil* or vocal cord*) adj5 (neoplas* or cancer* or carcinoma* or tumo?r*)).tw. |  |
| --- | --- |

| 4. ((gingival or leukoplakia or platal or partoid or sublingual or otorhinolaryn* or submandibular or ear or nose or maxillary sinus or orophar* or tracheal) adj5 (neoplas* or cancer* or carcinoma* or tumo?r*)).tw. |  |
| --- | --- |

| 5. Precancerous Conditions/ |  |
| --- | --- |

| 6. Erythroplasia/ |  |
| --- | --- |

| 7. exp Leukoplakia/ |  |
| --- | --- |

| 8. ((oral or mouth) adj dysplasia).tw. |  |
| --- | --- |

| 9. (leukoplakia or erythroplakia or preneoplas* or premalignant).tw. |  |
| --- | --- |

| 10. (precancerous or pre-cancerous or premalignant or pre-malignant).tw. |  |
| --- | --- |

| 11. 1 or 2 or 3 or 4 or 5 or 6 or 7 or 8 or 9 or 10 |  |
| --- | --- |

| 12. Smoking/ |  |
| --- | --- |

| 13. "Tobacco Use Disorder"/ |  |
| --- | --- |

| 14. smokeless tobacco.tw. |  |
| --- | --- |

| 15. chewing tobacco.tw. |  |
| --- | --- |

| 16. (smoking or smoker* or tobacco or nicotine or cigarette*).tw. |  |
| --- | --- |

| 17. 12 or 13 or 16 |  |
| --- | --- |

| 18. "Tobacco Use Cessation"/ or Smoking Cessation/ |  |
| --- | --- |

| 19. "Tobacco Use Cessation Products"/ |  |
| --- | --- |

| 20. Electronic Cigarettes/ |  |
| --- | --- |

| 21. smoking cessation.tw. |  |
| --- | --- |

| 22. (tobacco adj2 cessation).tw. |  |
| --- | --- |

| 23. (nrt or nicotine replacement or nicorette or niquitin or nicotinell).tw. |  |
| --- | --- |

| 24. (nicotine adj1 (gum* or inhaled or inhaler* or inhalator*)).tw. |  |
| --- | --- |

| 25. Propiophenones/ or Bupropion/ |  |
| --- | --- |

| 26. Acupuncture Therapy/ |  |
| --- | --- |

| 27. Acupuncture/ |  |
| --- | --- |

| 28. exp Anti-Anxiety Agents/ |  |
| --- | --- |

| 29. exp Antidepressive Agents/ |  |
| --- | --- |

| 30. Antidepressive Agents, Second-Generation/ |  |
| --- | --- |

| 31. Azocines/ |  |
| --- | --- |

| 32. Behavior Therapy/ |  |
| --- | --- |

| 33. Benzazepines/ |  |
| --- | --- |

| 34. Nicotinic Agonists/ |  |
| --- | --- |

| 35. Nicotinic Antagonists/ |  |
| --- | --- |

| 36. Nortriptyline/ |  |
| --- | --- |

| 37. Quinolizines/ |  |
| --- | --- |

| 38. Quinoxalines/ |  |
| --- | --- |

| 39. antidepressant*.tw. |  |
| --- | --- |

| 40. (antianxiety or anti-anxiety).tw. |  |
| --- | --- |

| 41. anxiolyti*.tw. |  |
| --- | --- |

| 42. bupropion.tw. |  |
| --- | --- |

| 43. clonidine.tw. |  |
| --- | --- |

| 44. cytisine.tw. |  |
| --- | --- |

| 45. dianicline.tw. |  |
| --- | --- |

| 46. doxepin*.tw. |  |
| --- | --- |

| 47. fluoxetin*.tw. |  |
| --- | --- |

| 48. imipramin*.tw. |  |
| --- | --- |

| 49. lazabemide.tw. |  |
| --- | --- |

| 50. lobeline.tw. |  |
| --- | --- |

| 51. mecamylamine.tw. |  |
| --- | --- |

| 52. moclobemide.tw. |  |
| --- | --- |

| 53. nicobrevin*.tw. |  |
| --- | --- |

| 54. (nicotine adj1 (gum* or inhaled or inhaler* or inhalator* or nasal or patch or therap* or vaccin* or replacement)).tw. |  |
| --- | --- |

| 55. nortryptiline.tw. |  |
| --- | --- |

| 56. paroxetine.tw. |  |
| --- | --- |

| 57. rimonabant.tw. |  |
| --- | --- |

| 58. selegilin*.tw. |  |
| --- | --- |

| 59. sertralin*.tw. |  |
| --- | --- |

| 60. silver acetat*.tw. |  |
| --- | --- |

| 61. tryptophan*.tw. |  |
| --- | --- |

| 62. vareniclin*.tw. |  |
| --- | --- |

| 63. venlafaxin*.tw. |  |
| --- | --- |

| 64. zimeledin*.tw. |  |
| --- | --- |

| 65. cessation aid.tw. |  |
| --- | --- |

| 66. ((quit* or stop* or abstain* or abstinence or ceas* or give up or treat* or therap*) adj1 (smoking or smoker* or tobacco or cigarette*)).tw. |  |
| --- | --- |

| 67. Counseling/ |  |
| --- | --- |

| 68. Directive Counseling/ |  |
| --- | --- |

| 69. Distance Counseling/ |  |
| --- | --- |

| 70. exp Exercise/ |  |
| --- | --- |

| 71. exp Hypnosis/ |  |
| --- | --- |

| 72. Patient Education as Topic/ |  |
| --- | --- |

| 73. acupuncture.tw. |  |
| --- | --- |

| 74. councel?ing.tw. |  |
| --- | --- |

| 75. counsel?ing.tw. |  |
| --- | --- |

| 76. exercise.tw. |  |
| --- | --- |

| 77. hypnosis.tw. |  |
| --- | --- |

| 78. patient education.tw. |  |
| --- | --- |

| 79. (behavio?r adj2 (change* or therap*)).tw. |  |
| --- | --- |

| 80. abstinen*.tw. |  |
| --- | --- |

| 81. psychologist*.tw. |  |
| --- | --- |

| 82. talking therap*.tw. |  |
| --- | --- |

| 83. psychotherap*.tw. |  |
| --- | --- |

| 84. ((cbt or cognitive) adj2 therap*).tw. |  |
| --- | --- |

| 85. exp Psychotherapy/ |  |
| --- | --- |

| 86. exp Cognitive Therapy/ |  |
| --- | --- |

| 87. 18 or 19 or 20 or 21 or 22 or 23 or 24 or 25 or 26 or 27 or 28 or 29 or 30 or 31 or 32 or 33 or 34 or 35 or 36 or 37 or 38 or 39 or 40 or 41 or 42 or 43 or 44 or 45 or 46 or 47 or 48 or 49 or 50 or 51 or 52 or 53 or 54 or 55 or 56 or 57 or 58 or 59 or 60 or 61 or 62 or 63 or 64 or 65 or 66 or 67 or 68 or 69 or 70 or 71 or 72 or 73 or 74 or 75 or 76 or 77 or 78 or 79 or 80 or 81 or 82 or 83 or 84 or 85 or 86 |  |
| --- | --- |

| 88. Alcohols/ |  |
| --- | --- |

| 89. exp Alcohol Drinking/ |  |
| --- | --- |

| 90. exp Alcohol-Related Disorders/ |  |
| --- | --- |

| 91. Alcoholism/ |  |
| --- | --- |

| 92. alcohol*.tw. |  |
| --- | --- |

| 93. (alcohol adj2 (abuse* or misuse* or user* or disorder* or consumption or drink*)).tw. |  |
| --- | --- |

| 94. ((heavy or binge or risk* or hazard*) adj drinking).tw. |  |
| --- | --- |

| 95. 88 or 89 or 90 or 91 or 92 or 93 or 94 |  |
| --- | --- |

| 96. Psychotherapy, Brief/ |  |
| --- | --- |

| 97. ((alcohol or drink*) adj2 (abstain or abstin* or give up or reduce or cease or cessation or reduction or intervention)).tw. |  |
| --- | --- |

| 98. ((early or brief or minimal) adj intervention).tw. |  |
| --- | --- |

| 99. 96 or 97 or 98 |  |
| --- | --- |

| 100. exp Randomized Controlled Trials as Topic/ |  |
| --- | --- |

| 101. Random Allocation/ |  |
| --- | --- |

| 102. Double-Blind Method/ |  |
| --- | --- |

| 103. Single-Blind Method/ |  |
| --- | --- |

| 104. Clinical Trial/ |  |
| --- | --- |

| 105. exp Clinical Trials as Topic/ |  |
| --- | --- |

| 106. clinical trial, phase i.pt. |  |
| --- | --- |

| 107. clinical trial, phase ii.pt. |  |
| --- | --- |

| 108. clinical trial, phase iii.pt. |  |
| --- | --- |

| 109. clinical trial, phase iv.pt. |  |
| --- | --- |

| 110. controlled clinical trial.pt. |  |
| --- | --- |

| 111. randomi#ed controlled trial.pt. |  |
| --- | --- |

| 112. multicenter study.pt. |  |
| --- | --- |

| 113. clinical trial.pt. |  |
| --- | --- |

| 114. (clinical adj trial).tw. |  |
| --- | --- |

| 115. ((sing* or doubl* or treb* or tripl*) adj (blind* or mask*)).tw. |  |
| --- | --- |

| 116. Placebos/ |  |
| --- | --- |

| 117. placebo*.tw. |  |
| --- | --- |

| 118. exp Placebo Effect/ |  |
| --- | --- |

| 119. random*.tw. |  |
| --- | --- |

| 120. (crossover or cross-over).tw. |  |
| --- | --- |

| 121. RCT*.tw. |  |
| --- | --- |

| 122. ((allocat* or assign*) adj2 random*).tw. |  |
| --- | --- |

| 123. (randomi?ed or randomi?ation).tw. |  |
| --- | --- |

| 124. randomly.ab. |  |
| --- | --- |

| 125. ((administ* or allocat* or assign* or class* or control* or determine* or divide* or distribut* or expose* or fashion* or number* or place* or recruit* or substitut* or treat*) adj3 random*).tw. |  |
| --- | --- |

| 126. drug therapy.fs. |  |
| --- | --- |

| 127. trial.tw. |  |
| --- | --- |

| 128. groups.ab. |  |
| --- | --- |

| 129. (control* adj3 (trial* or study or studies)).tw. |  |
| --- | --- |

| 130. ((singl* or doubl* or treb* or tripl*) adj3 (blind* or mask* or dummy*)).tw. |  |
| --- | --- |

| 131. (control* adj3 (trial* or study or studies)).tw. |  |
| --- | --- |

| 132. ((singl* or doubl* or treb* or tripl*) adj3 (blind* or mask* or dummy*)).mp. |  |
| --- | --- |

| 133. Clinical Trial, Phase II/ |  |
| --- | --- |

| 134. Clinical Trial, Phase III/ |  |
| --- | --- |

| 135. Clinical Trial, Phase IV/ |  |
| --- | --- |

| 136. exp Randomized Controlled Trial/ |  |
| --- | --- |

| 137. exp Pragmatic Clinical Trial/ |  |
| --- | --- |

| 138. ((waitlist* or wait* list* or treatment as usual or TAU) adj3 (control or group)).ab. |  |
| --- | --- |

| 139. 96 or 97 or 98 or 99 or 100 or 101 or 102 or 103 or 104 or 105 or 106 or 107 or 108 or 109 or 110 or 111 or 112 or 113 or 114 or 115 or 116 or 117 or 118 or 119 or 120 or 121 or 122 or 123 or 124 or 125 or 126 or 127 or 128 or 129 or 130 or 131 or 132 or 133 or 134 or 135 or 136 or 137 or 138 |  |
| --- | --- |

| 140. animals/ not humans/ |  |
| --- | --- |

| 141. exp Animals, Laboratory/ |  |
| --- | --- |

| 142. exp Animal Experimentation/ |  |
| --- | --- |

| 143. Models, Animal/ |  |
| --- | --- |

| 144. Rodentia/ |  |
| --- | --- |

| 145. (rat* or mouse or mice).ti. |  |
| --- | --- |

| 146. 140 or 141 or 142 or 143 or 144 or 145 |  |
| --- | --- |

| 147. (child* or infant* or girl* or boy* or teenager* or adolescen* or baby or babies or newborn*).tw. |  |
| --- | --- |

| 148. 17 or 95 |  |
| --- | --- |

| 149. 87 or 99 |  |
| --- | --- |

| 150. 11 and 139 and 148 and 149 |  |
| --- | --- |

| 151. 146 or 147 |  |
| --- | --- |

| 152. 150 not 151 |
| --- |
